# Diversity partitioning in Phanerozoic benthic marine communities

**DOI:** 10.1073/pnas.1814487116

**Published:** 2018-12-17

**Authors:** Richard Hofmann, Melanie Tietje, Martin Aberhan

**Affiliations:** ^a^Museum für Naturkunde Berlin, Leibniz Institute for Evolution and Biodiversity Science, 10115 Berlin, Germany

**Keywords:** biodiversity, biotic interactions, beta diversity, alpha diversity, paleoecology

## Abstract

Biotic interactions such as competition, predation, and niche construction are fundamental drivers of biodiversity at the local scale, yet their long-term effect during earth history remains controversial. To test their role and explore potential limits to biodiversity, we determine within-habitat (alpha), between-habitat (beta), and overall (gamma) diversity of benthic marine invertebrates for Phanerozoic geological formations. We show that an increase in gamma diversity is consistently generated by an increase in alpha diversity throughout the Phanerozoic. Beta diversity drives gamma diversity only at early stages of diversification but remains stationary once a certain gamma level is reached. This mode is prevalent during early- to mid-Paleozoic periods, whereas coupling of beta and gamma diversity becomes increasingly weak toward the recent. Generally, increases in overall biodiversity were accomplished by adding more species to local habitats, and apparently this process never reached saturation during the Phanerozoic. Our results provide general support for an ecological model in which diversification occurs in successive phases of progressing levels of biotic interactions.

## Significance

Biotic interactions are drivers of biodiversity, yet their effects on Phanerozoic marine diversity remain elusive because they operate on small spatial scales. We provide the comprehensive reconstruction of within-community, between-community, and overall diversity on the scale of geological formations throughout the Phanerozoic eon to gauge the effects of biotic interactions on biodiversity. Within-community and overall diversity are positively correlated and both are practically unbounded. Between-community diversity drives overall diversity only at low levels of overall diversity, and mostly during the early- to mid-Paleozoic. Further increase of biodiversity is generally achieved by finer resource partitioning driven by positive species interactions.

Deciphering biodiversity patterns and their drivers for the geological past attracts unabated interest (1, 2) to identify the principal processes of diversification and the response of the biosphere to environmental perturbations (3). Species interactions such as competition, predation, and niche construction are key to the understanding of diversity accumulation and potential saturation effects in local and regional species richness (4–6). These aspects are highly scale-dependent but might be unmasked by testing hypotheses at the geographic and temporal scale on which they are expected to operate. Here, we apply the concept of diversity partitioning to the fossil record to unveil the role of biotic controls in diversification and to examine potential limits to local biodiversity. Introduced by Whittaker (7, 8), biodiversity can be dissected into three components known as alpha diversity (local species richness), beta diversity (differential diversity between localities), and gamma diversity (overall species richness of the observed system). This concept proved useful to disentangle patterns underlying diversification in modern biota (9, 10). Some applications of diversity partitioning in deep time exist (11, 12) but are confronted with the problem that biotic factors and abiotic factors (e.g., plate configuration, changes in physicochemical conditions) increasingly intermingle from local to regional and global scales (13, 14). Apart from a few studies at short timescales (15, 16), the structure and limits of Phanerozoic biodiversity at the habitat scale are largely unknown.

We used occurrence data of noncolonial benthic marine invertebrate species (gastropods, bivalves, trilobites, brachiopods, and echinoderms) from the Paleobiology Database (https://paleobiodb.org) to determine alpha, beta, and gamma diversity of 340 Phanerozoic geological formations (see *Materials and Methods* for details and *SI Appendix*, Fig. S1). Formations are mappable geological units of mostly sedimentary rock that usually maintain a more or less uniform environmental architecture during a certain time span at a given place in the geological past. As such, marine formations harbor a pool of species that were principally able to interact at the local and regional scale and thus can be regarded as the constituents of metacommunities in the geological record. Beta diversity among collections from the same formation is expected for two reasons, even though many benthic marine invertebrates have planktonic larval stages and thus high dispersal capabilities. First, the distribution of species within a metacommunity is predicted to be patchy (17). Even if the habitats represented by a formation were homogeneous, local differences in species distributions would result in compositional differences among sites. Beta diversity is, thus, expected to structure gamma diversity also under neutral conditions (17). Second, the formations analyzed herein typically capture more than just one habitat (*SI Appendix*, Fig. S2) and thereby record environmental differentiation. Accordingly, we expect differences in faunal composition, and therefore beta diversity. Fossil collections are usually time-averaged, i.e., they constitute a mix of skeletal elements of noncontemporaneous communities as a result of taphonomic and sedimentological processes (18). Compared with the former live assemblages, time averaging enhances alpha diversity and diminishes beta diversity in death assemblages and fossil assemblages (18–20). However, time averaging does not completely eliminate original beta diversity (20) and, respective gradients in species composition are still conserved in death assemblages (21). Collectively, we therefore expect beta diversity to be conveyed by the fossil collections of the formations studied herein.

Utilizing formations allows us to test the significance of biologically controlled diversity partitioning, particularly the role of species interactions, by reducing purely abiotic effects and artifacts (e.g., provinciality, tectonic configuration, preserved rock volume per time interval, binning of time intervals) that are known to influence global diversity estimates (22, 23). This approach allows us to test the general expectations of ecological diversification models that are based on positive biotic interactions, i.e., processes such as competition, predation, and niche construction that promote diversity. In particular, we test the theoretical diversity partitioning model of Hautmann (24) in which pathways of alpha and beta diversity were used to define three phases of diversity accumulation mediated by progressively increasing levels of positive interactions (see *Discussion* for details). In this study, alpha is the mean number of species per collection, beta is the mean dissimilarity between collections in each stratigraphic formation, and gamma is the total species richness within each formation.

## Results

The trajectories of mean alpha and mean gamma diversity in formations show a largely similar course during the Phanerozoic (*SI Appendix*, Fig. S3 *A* and *B*). Aside from peaks in the Silurian and Permian, both diversity metrics show no pronounced trend until the mid-Cretaceous. Since the late Cretaceous they increase on average toward the recent. Beta diversity, measured using Simpson-based multiple-site dissimilarity (25) (named as Simpson’s metric below), and Whittaker’s (8) beta show no obvious trend throughout the Phanerozoic (*SI Appendix*, Fig. S3 *C* and *D*). Immediate effects of mass extinctions on any diversity measure are not readily discernible from the plots because of the coarse temporal resolution.

**Fig. 1.**
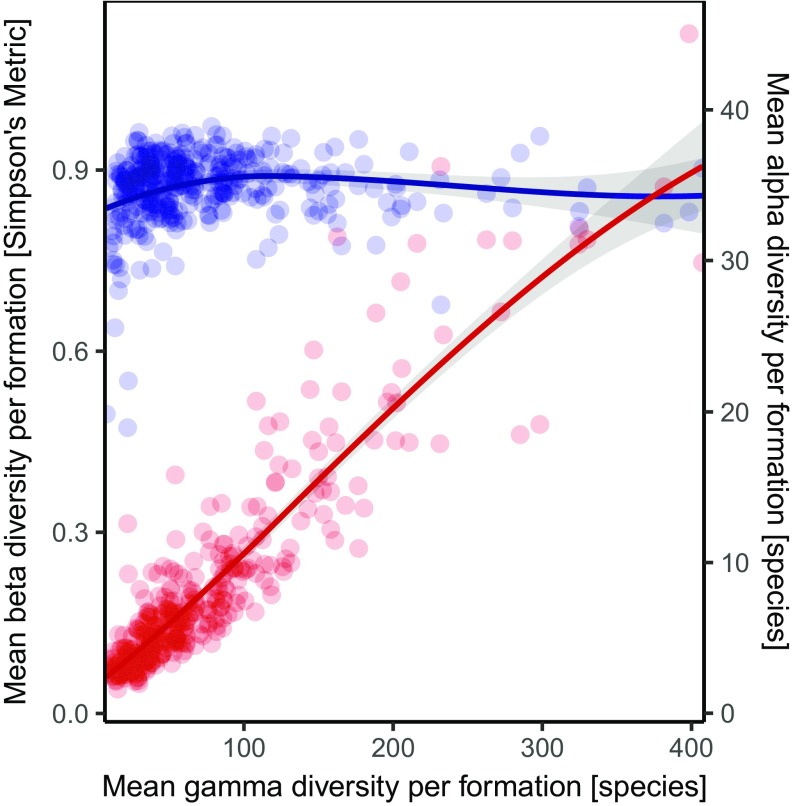
Alpha–beta–gamma-plot showing the influence of alpha (red) and Simpson’s metric as a measure for beta diversity (blue) to generate gamma diversity of all Phanerozoic geological formations. Gray areas represent the 95% confidence intervals of curve fits (Loess fit, bold lines). Gamma diversity change is proportional to alpha diversity (red line). Beta diversity (blue line) appears to have a neutral relationship to gamma diversity. *Y* axes refer to mean species richness of the collections of a formation (i.e., alpha diversity) and to Simpson’s metric, respectively.

**Fig. 2.**
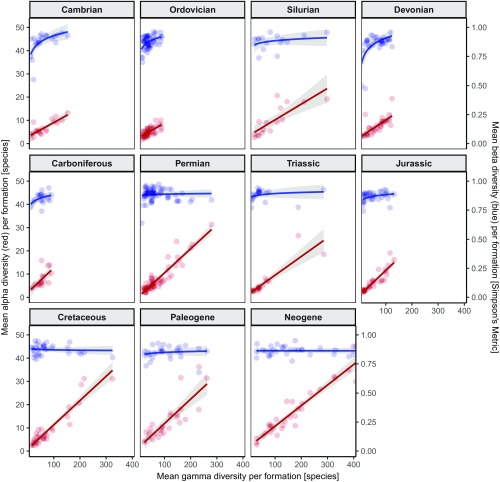
Alpha–beta–gamma plots showing the contributions of alpha diversity (red) and mean Simpson’s metric as a measure of beta diversity (blue) to generate gamma diversity for each formation split by geological period. Gray areas represent the 95% confidence intervals of curve fits. Logarithmic fits for beta diversity and linear fits for alpha diversity provided best representations of the data (*SI Appendix*, Fig. S11). Note uniform scale for all panels.

High gamma diversity can be gained by increasing alpha diversity, beta diversity, or both. Alpha–beta-gamma–plots (24, 26) visualize the generation of gamma diversity in relation to alpha and beta diversity (Fig. 1). We find a strong and consistent positive relationship between alpha and gamma (Spearman’s rho = 0.87, *P* << 0.01), indicating that high species richness of formations is consistently related to high species richness of the constituent local communities. By contrast, the contribution of beta diversity to changes in gamma diversity is fairly uniform. For the Phanerozoic as a whole, species-rich formations do not tend to have higher differential diversity between collections than formations with less species (Fig. 1). When beta–gamma plots are constructed for separate time periods (Fig. 2), some time intervals show a “low- beta-first” trajectory (e.g., the Cambrian, Ordovician, Devonian, and to some extent the Carboniferous). These fits indicate a positive relationship between Simpson’s metric and gamma diversity. More species-rich periods (e.g., the Silurian, Triassic) which still exhibit this low-beta-first signal show that this relationship weakens toward high gamma diversity when beta levels off (see also *SI Appendix*, Fig. S4). Some other periods (e.g., the Cretaceous, Neogene) show a more stationary Simpson’s metric across a wider range of gamma, which suggests a high-beta-first mode of diversity assembly.

## Discussion

The positive and consistent correlation between alpha and gamma diversity of formations suggests that species can be packed into fairly small-scale habitats with no perceivable upper limit. If “saturation” had occurred in alpha diversity, its trajectory would level off in alpha–gamma–plots which is not the case (Figs. 1 and 2). If there were limits to alpha (and gamma) diversity, they were never reached during the Phanerozoic (5, 27). Remarkably, alpha and gamma diversity are escalating since the late Mesozoic with no obvious limits (*SI Appendix*, Fig. S3 *A* and *B*). A probable explanation is provided by the principal reorganization of marine ecosystems referred to as the “Mesozoic Marine Revolution” (28). Its hallmarks––intensified predation, infaunalization, and a general expansion in ecospace utilization (29)––increased the total carrying capacity of marine ecosystems, which, from then on, was even less likely to be reached (5). This general increase in positive interactions during the late Mesozoic represents a plausible mechanism to unlock species diversity in benthic marine communities.

Beta diversity, as expressed by the Simpson’s metric, has inherent boundaries ranging from 0 (collections are identical in species composition) to 1 (collections do not share a single species). Beta diversity is not randomly distributed across the whole spectrum of gamma diversity. Fig. 2 shows that the strength of coupling between the two variables depends on overall (gamma) diversity as beta diversity levels off at high gamma, marking the maximum taxonomic differentiation among the collections of a formation. These upper levels of beta diversity are higher in the early- and mid-Paleozoic and tend to decrease toward the recent (*SI Appendix*, Figs. S4 and S5). Furthermore, low-beta-first fits appear to be more prevalent during the lower part of the Paleozoic (Fig. 2 and *SI Appendix*, Fig. S4), which could suggest that the coupling between beta and gamma (at low gamma diversity) is stronger during these times than later on.

To test this hypothesis, we constructed a time-independent null model for beta–gamma fits in which formations are randomly drawn from the overall data pool (*Materials and Methods* and *SI Appendix*, Fig. S6). To allow for an unbiased comparison among time intervals, we restricted the data to the range of gamma diversity over which possible beta dependence occurs. The upper threshold of this range lies at a gamma diversity of ∼100 species (Fig. 2 and *SI Appendix*, Fig. S4). Apart from a very weak trend toward a decreasing strength of beta–gamma coupling across the Phanerozoic (*SI Appendix*, Fig. S7), the lower part of the Paleozoic is not different on a significant level than in younger time intervals in this respect (*SI Appendix*, Fig. S7). We conclude that there is no principal difference in the mode of diversity assembly. Beta–gamma trajectories are mainly controlled by the maximum gamma diversity that is reached in respective time periods rather than by genuine changes in beta diversity.

**Fig. 3.**
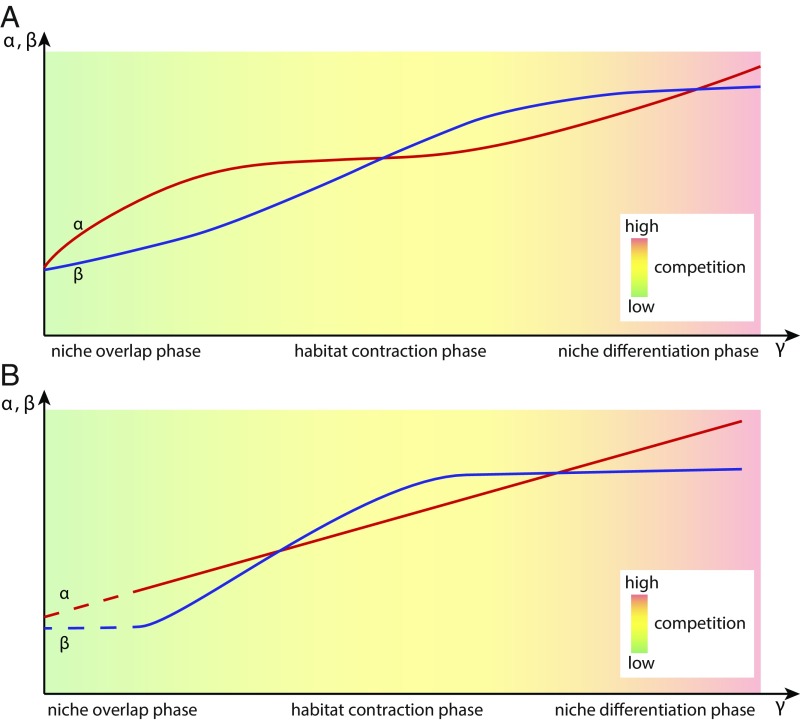
Hautmann’s (24) multiplicative diversification model showing alpha and beta diversity as a function of gamma diversity (α–β–γ plots). α, alpha diversity; β, beta diversity; γ, gamma diversity. (*A*) Original model. (*B*) Adjusted empirical model accounting for the findings presented herein. Dashed lines for α and β indicate that this phase is not recorded.

Pathways of alpha and beta diversity were used in the diversification model of Hautmann (24) to define three phases of diversity accumulation mediated by progressively increasing levels of positive interactions (Fig. 3). The first stage is the niche overlap phase in which generally few species exploit a wide range of habitats owing to a low rate of diffuse competition (30). This phase is characterized by low beta diversity and low but increasing alpha diversity. It is followed by the habitat contraction phase. With rising alpha diversity, emerging interspecific competition forces species toward their ecological optima and thereby fosters habitat specialization reflected in increasing beta diversity. In the final niche differentiation phase, the maximum degree of habitat differentiation is realized and reflected in high but stagnant beta diversity. A further increase in gamma diversity can only be achieved by finer partitioning of local niche space expressed in higher alpha diversity. The late niche overlap and the habitat contraction phase are herein identified in periods in which increasing beta diversity contributes to a rise in gamma diversity until a certain gamma level is reached (most notably the Cambrian, Ordovician, and Devonian, Fig. 2). Time intervals which reach higher levels of gamma diversity, and in which beta levels off (Silurian, Permian, Fig. 2), represent the habitat contraction and the early niche differentiation phase (Fig. 3*A*). The Silurian and particularly the Permian thus represent Paleozoic climaxes of niche differentiation within geological formations, with high values of beta and alpha diversity (*SI Appendix*, Fig. S3). Periods in which beta is mostly invariant (e.g., the Cretaceous, Neogene, and to some extent the Paleogene, Fig. 2) are best explained by the niche differentiation phase without a perceivable habitat contraction phase taking place beforehand. The early niche overlap phase is not observed, most likely because candidate time intervals [e.g., the Early Triassic (31, 32)] are too short, to emerge in our compilation. The distribution of the three phases of the Hautmann model during the Phanerozoic roughly corresponds with the trajectory of global genus diversity (1). The late stage of the habitat contraction and the habitat differentiation phase mirror peaks in global diversity whereas the early habitat contraction phase prevails during times with reduced global diversity. Further analyses will have to reveal how the overall diversity of formations and beta diversity among formations, which was not analyzed in this study, interacted to generate global Phanerozoic diversity.

Overall, our results largely support Hautmann’s diversification model with one notable deviation (Fig. 3*B*): There is no apparent change in the strength of coupling between alpha and gamma diversity, which could be used to make a distinction between the niche overlap and the habitat contraction phase. To the extent that increasing beta diversity in formations conveys a biotic signal, we suggest that positive interactions drive diversification at low and intermediate levels of gamma diversity. The observation that marine faunal specialization increases with overall diversity (6) provides additional independent evidence that diversification is, at least temporarily, driven by ecosystem differentiation. Moreover, the proportion of specialists in global compilations (6) is largely tracked by our beta diversity time series (*SI Appendix*, Fig. S3 *C* and *D*). The identified maxima in taxonomic differentiation (Fig. 2 and *SI Appendix*, Fig. S3) suggest that there is a saturation point with respect to beta diversity beyond which further diversity can only be gained by adding more species to local habitats. As most periods exhibit similar diversity trajectories (Fig. 2 and *SI Appendix*, Fig. S4), we posit that the slightly modified diversification model (Fig. 3*B*) can be universally applied to Phanerozoic marine benthic ecosystems.

## Materials and Methods

### Data.

The workflow of the study is illustrated in *SI Appendix*, Fig. S1. Species occurrence matrices were retrieved from the Paleobiology Database in April 2017 via fossilworks.org/. Included taxa of the marine macrobenthos comprise Trilobita, Brachiopoda, Echinodermata, Gastropoda, and Bivalvia as they constitute the majority of level bottom communities during the Phanerozoic. Separate matrices were constructed for each geological period. The following items were downloaded for each collection: species composition, formation name, midage, reference ID, depositional environment, and geographic paleocoordinates. Environmental categories were standardized by assigning inconsistently used terms to the following categories: reefal, deep subtidal, shallow subtidal, marginal marine, slope, basin, and unknown. The data vetting procedure for each matrix included, where applicable, revisions of formation names, taxonomic names, and age assignments. Taxa with unclear genus identification were discarded, and so were collections lacking formational assignment. If questionable ages appeared (e.g., ages notably deviating from ages of other collections from the same formation), they were either revised (using referenced literature) or excluded. Collections with just one recorded taxon were omitted. After vetting, only formations with more than 25 collections were included in the analysis. Including formations with fewer collections would have reduced the respective subsampling level, which would have lowered the reliability of the reconstructed diversity measures. These vetted matrices were the basis for further processing with R (33).

### Subsampling and Plotting of Data.

Determining diversity may be prone to biases. We used a subsampling routine to reduce biases stemming from varying sampling intensity which is the main source for uneven diversity estimates in composite datasets. A submatrix for each formation was extracted from the matrices of the geological periods. This formational matrix was then subsampled by randomly drawing 20 collections without replacement. This procedure was repeated 500 times for each formation. In each subsampled matrix, alpha, gamma, and several measures of beta diversity were calculated. Alpha diversity represents the average number of species per collection. Gamma diversity represents the overall number of species of a formation as recorded by the subsampled matrix. We used the Simpson-based multiple-site dissimilarity (25): here Simpson’s metric or *BetaSim* in scripts) to get an independent estimate of beta diversity. This multisite index takes into account the “nestedness” of ecological (and paleontological) samples. Different samples, for example, often represent subsets of a larger association of taxa (paleocommunity or metacommunity). The lack of taxa rather than the record of different taxa (i.e., turnover) thus may produce much of the dissimilarity recovered using other beta indices. Simpson’s metric emphasizes turnover rather than lack of taxa and thus circumvents problems typically associated with the nature of paleontological samples (see also ref. 34). The classic beta diversity measure known as Whittaker’s beta (*BetaW*) represents the ratio of gamma and average alpha. It has been calculated because of its wide use in the literature. All these measures were averaged from the 500 trials. The final table upon which graphic and statistical analyses were performed contains these mean values for each formation (*AlphaForm, BetaWForm, BetaSimForm, GammaForm*) next to its metadata including: name (*formation.name*), age (average midage in million years), geological period (*Period*), duration (maximum midage - minimum midage in million years), number of collections per formation (*CollpF*, ultimate number of collections pertaining to one formation), number of environments (*Environments*, number of different environmental categories recorded in each formation), and references per formation (*RefForm*, number of references contributing to formation matrix). The maximum and median great circle distance (*maxGCD* and *medianGCD*) as well as the median absolute deviation based on paleocoordinates of collections for each formation was calculated as a measure for its spread. All figures were produced with the R (33) plotting package *ggplot2*. Calculations were made using the packages beta.part (35) and vegan (36). All R scripts, downloaded matrices, and Paleobiology Database references are available at https://github.com/fossilrich/Diversity-Partitioning.

### Null Model Testing.

We constructed a null model (*SI Appendix*, Fig. S6) to provide a framework for testing the significance (*SI Appendix*, Fig. S7) of potentially different diversity trajectories (Fig. 2) by drawing 20 formations at random from the Phanerozoic data pool. We constructed fits of alpha and beta diversity for each trial and compared the average slope (beta–gamma coupling) and its SD from 200 trials against the actual slopes of the individual periods (*SI Appendix*, Fig. S7).

### Statistical Tests.

Using Spearman rank correlations, we tested whether or not potentially confounding factors (variation in numbers of references, collections, and environments per formation, duration of formations, number of higher taxa present, see also *SI Appendix*, Fig. S8) had an effect on diversity estimates after subsampling. Correlations between these factors and diversity are weak (*SI Appendix*, Table S1). One exception is the number of published studies that contribute to the formation matrix which correlates with estimates of gamma and beta diversity (rho values ∼ 0.4, *P* << 0.01). Taxonomic studies are often provided as “monophyletic” contributions. This bears the risk that fossil material from even the same bed enters the Paleobiology Database (PBDB) as different collections. This might create artificially high beta diversity and depresses alpha diversity. To test for this monographic effect, we discarded all formations with more than 10 references. This was the threshold below which Spearman tests yielded lowest correlation coefficients for number of references to influence diversity (*SI Appendix*, Table S1). Analyses of this modified dataset produces identical alpha–beta–gamma plots (*SI Appendix*, Figs. S9 and S10). This indicates that a supposed monographic effect does not distort the overall signal. The Simpson’s metric (25) strongly correlates with Whittaker’s beta diversity (Spearman rank correlation for Beta_Sim_ rho = 0.89, *P* < 0.01).

### Data Access and Availability.

All data and codes used to conduct analyses and plot figures are available from GitHub at https://github.com/fossilrich/Diversity-Partitioning.

## Supplementary Material

Supplementary File
