# Supplementary material for: Diversity partitioning in Phanerozoic benthic marine communities
Source: Proc Natl Acad Sci U S A. 2018 Dec 17;116(1):79–83. doi: 10.1073/pnas.1814487116 (PMC6320541; doi:10.1073/pnas.1814487116)
Supplement: Supplementary File [file pnas.1814487116.sapp.pdf]

Supplementary Information

to accompany the manuscript

Diversity partitioning in Phanerozoic benthic marine communities

Richard Hofmann*, Melanie Tietje, Martin Aberhan

*Corresponding author. E-mail: richard.hofmann@mfn-berlin.de

This file includes:

Figs. S1 to S11

Table S1

www.pnas.org/cgi/doi/10.1073/pnas.1814487115

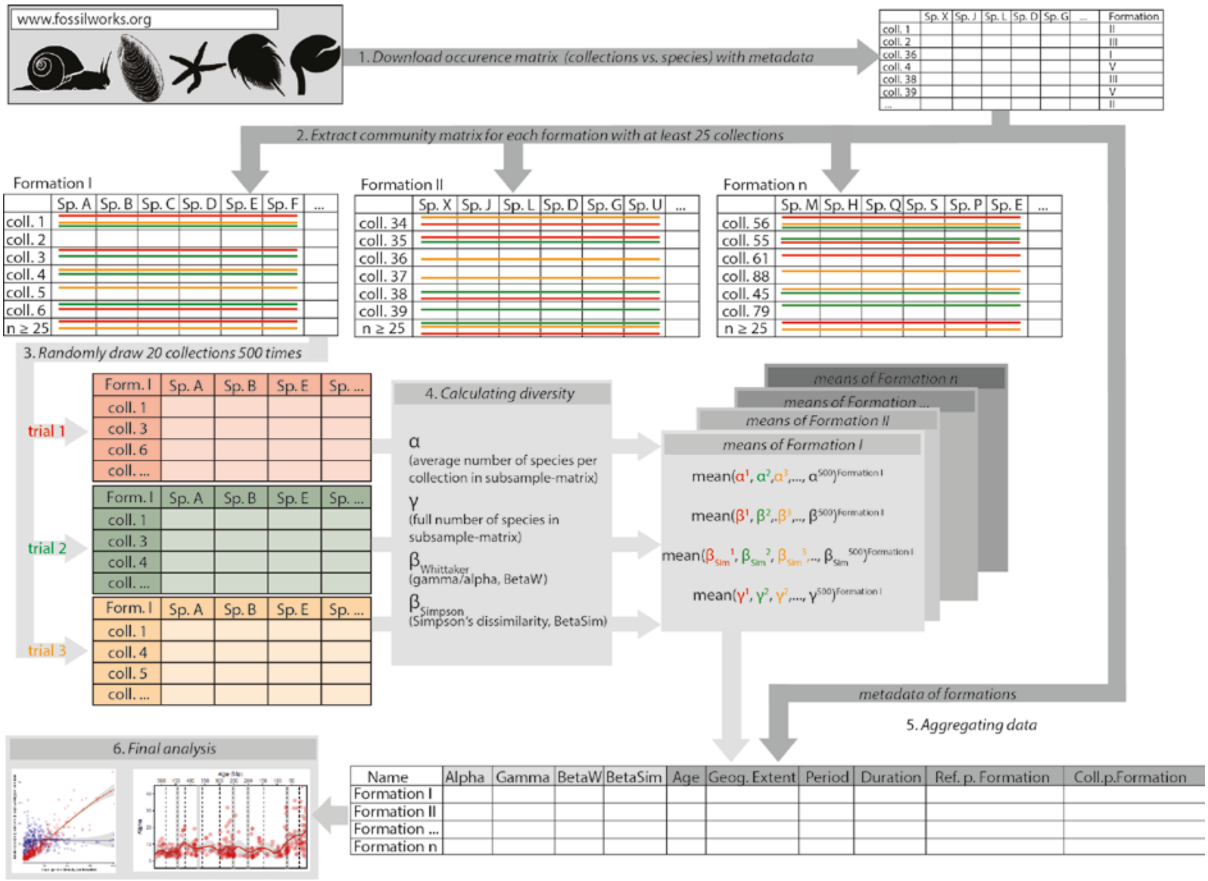

Fig. S1. Workflow of the analysis applied herein. Colored lines in formation matrices illustrate different collections to be drawn at each trial.

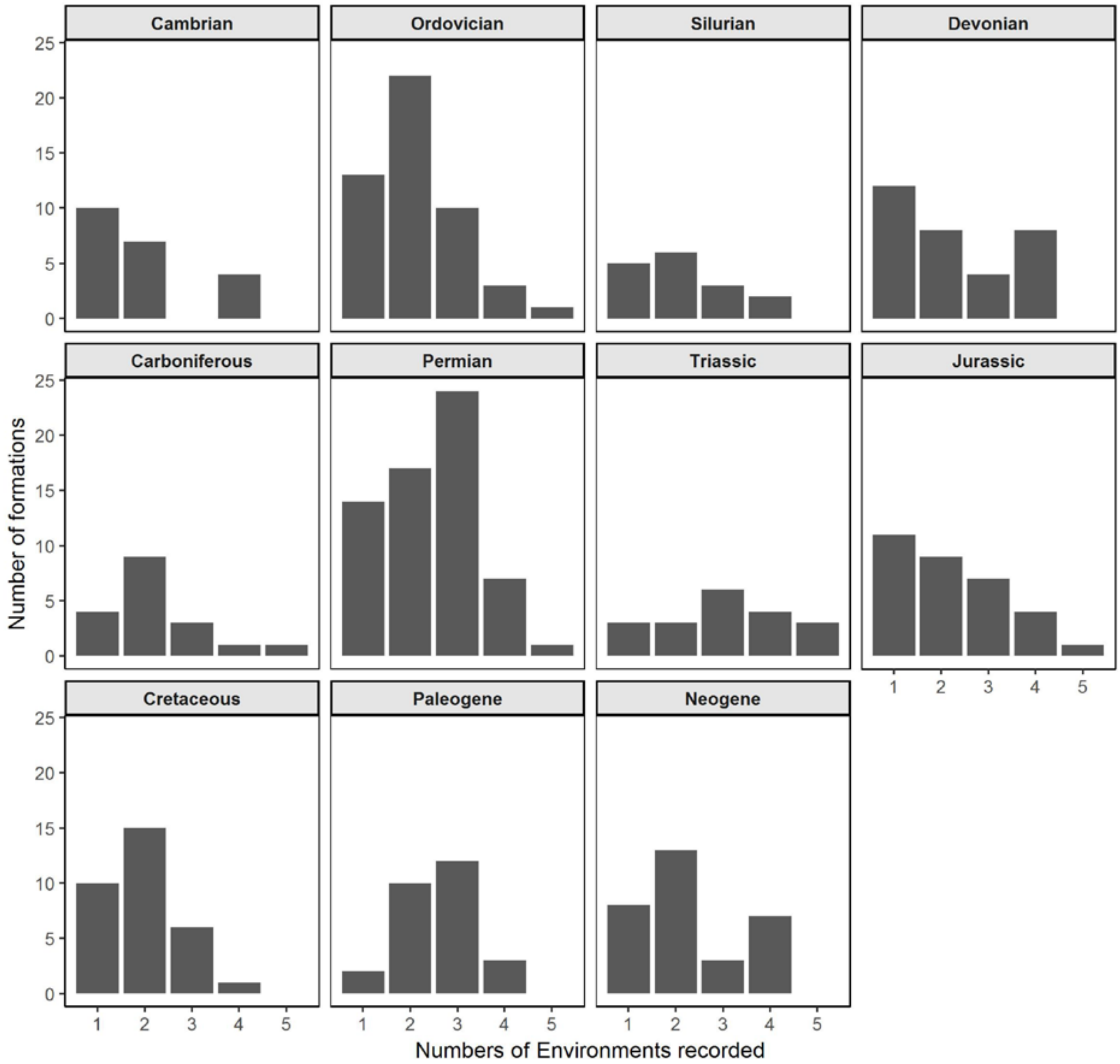

Fig. S2. Barplots showing the number of environments recorded in formations from each time interval.

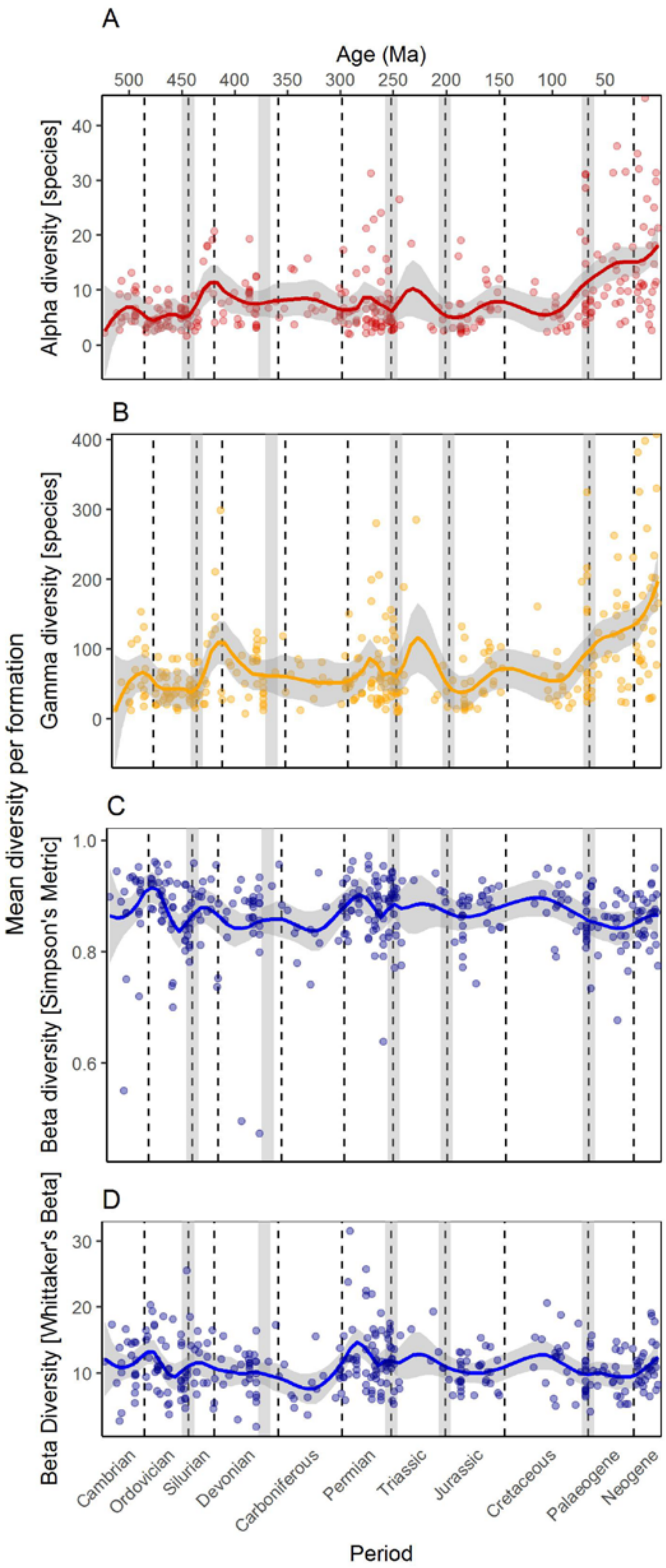

Fig. S3. Time series plots for alpha, beta and gamma diversity for 331 Phanerozoic formations retrieved from the Paleobiology Database. Dashed lines represent boundaries of geological periods, grey vertical bars indicate the “Big five” mass extinction events. Bold lines represent loess fit curves with 95% confidence intervals in grey. (**A**) Mean alpha diversity (mean species richness of collections). (**B**) Mean gamma diversity (mean species richness of all collections of a formation combined). (**C**) Mean Simpson’s Metric (after Baselga 2010). (**D**) Mean Whittaker’s beta diversity (i.e. the ratio of mean gamma diversity and mean alpha diversity).

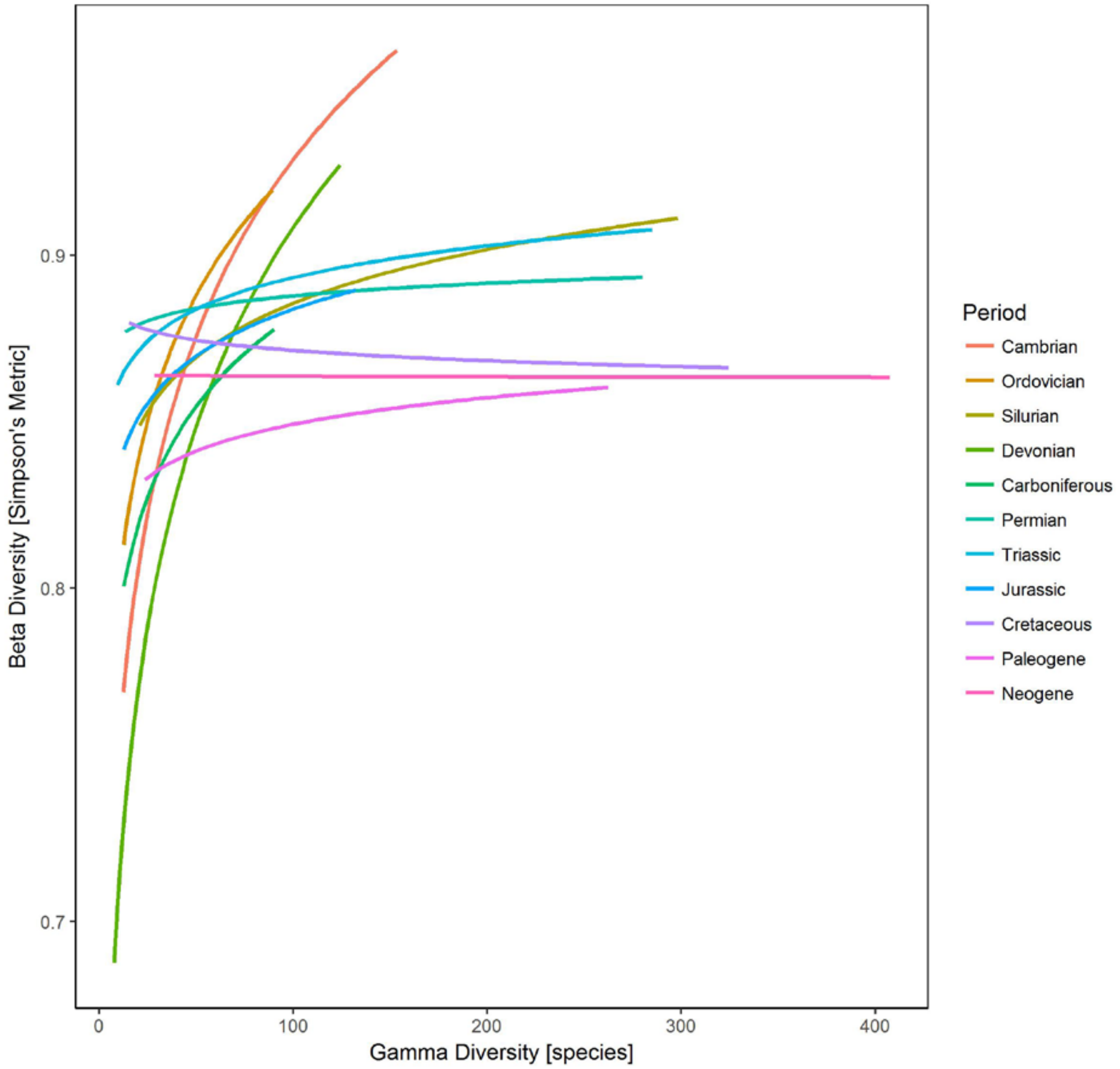

Fig. S4. Fits to beta diversity of each geological period of Figure 2 plotted in one graph.

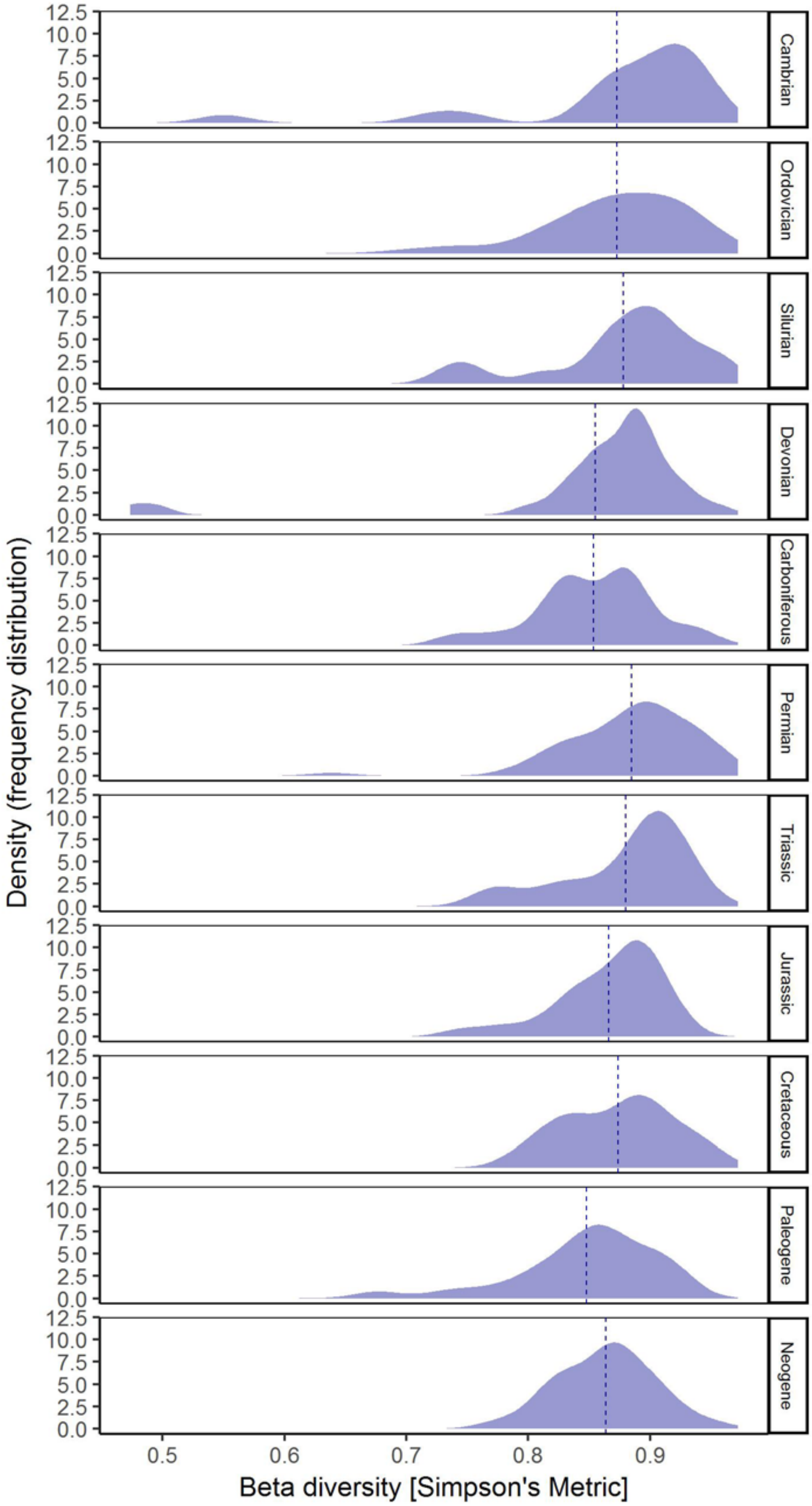

Fig. S5. Density plots of beta diversity for each geological period. Means shown by dashed line.

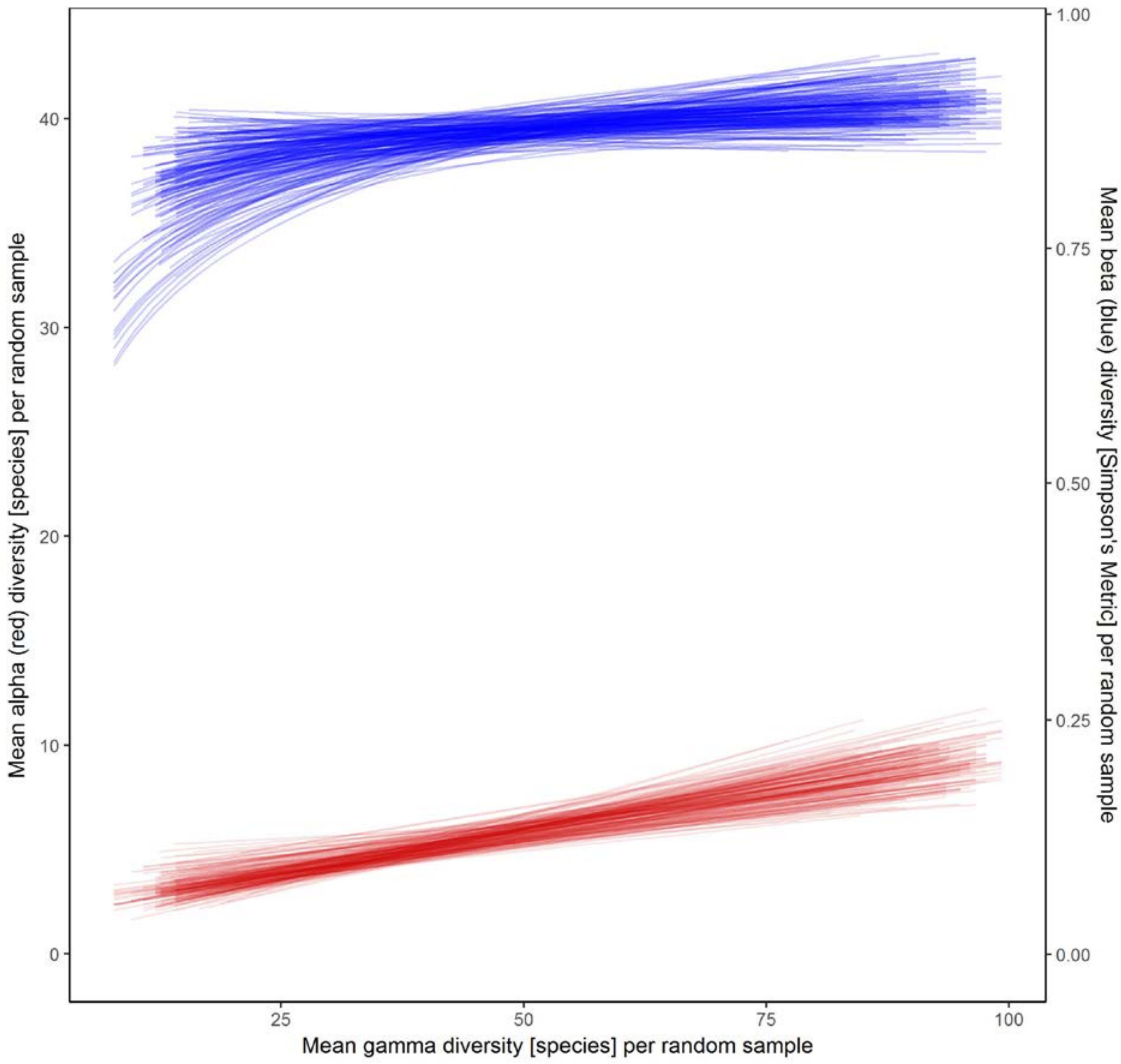

Fig. S6. Global Null model of fits of alpha and beta diversity from 200 random samplings from Formations with less than 100 species in gamma diversity.

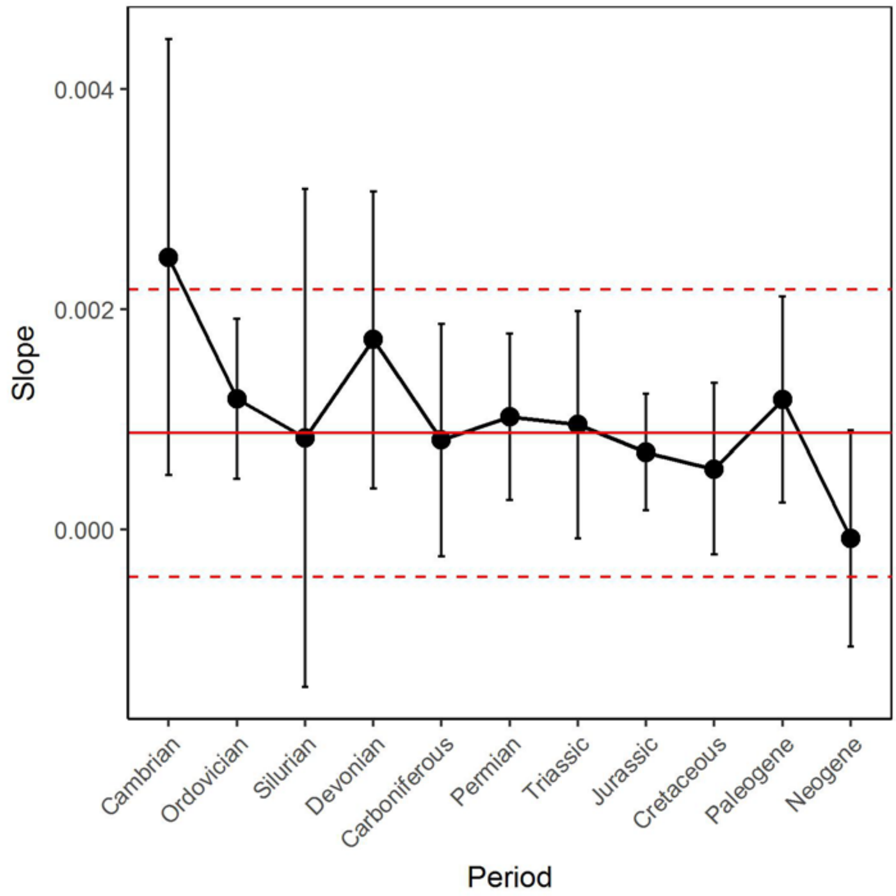

Fig. S7. The slopes of linear models fitted to the beta-gamma-plots compared to the mean (solid red line) and double standard deviation (dashed red lines) of the Null model slope for each period obtained from formation with less than 100 species in gamma diversity.

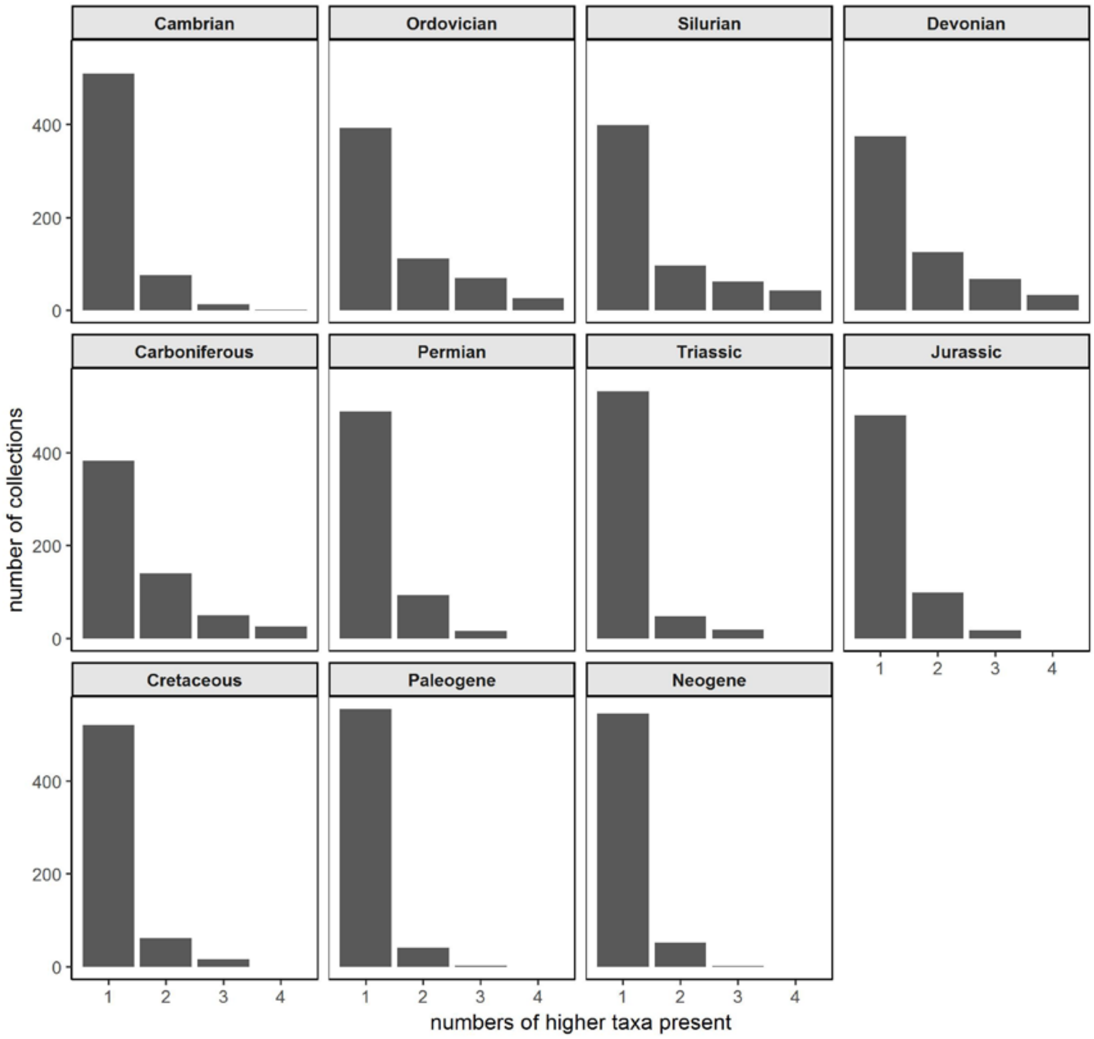

Figure S8: Barplots showing the number of higher taxa recorded in collections from each time interval. Subsample level is 600 random draws (lowest number of collections Silurian = 686). It shows that the portion of collections with 1, 2, 3, and 4 higher taxa, respectively, is not principally different on a Phanerozoic scale: Beds that are "monotaxic" are the most abundant ones in most time intervals. The only difference is that younger samples become less "diverse" mainly as a result of the post-Paleozoic absence of trilobites and the progressing demise of brachiopods since the Cretaceous. A potentially elevation of beta diversity due to supposed heterogeneity is, thus, rather unlikely. Additionally, the number of higher taxa recorded in each formation has no effect on any of the diversity measures (Table S1).

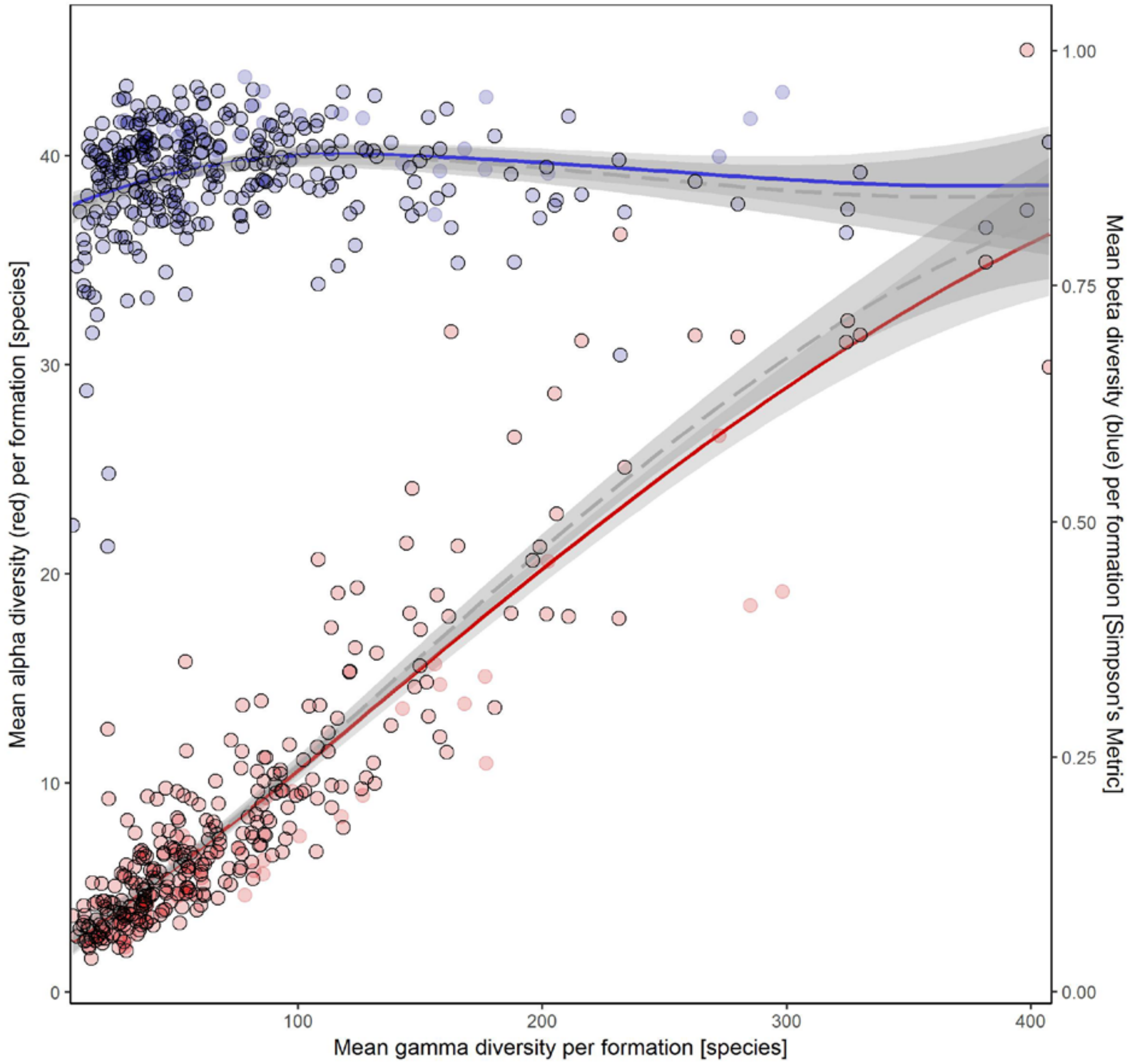

Fig. S9. Comparison of Phanerozoic alpha-beta-gamma-plot of the full data set (all 340 recognized formations: unlined dots and dashed fit) with the alpha-beta-gamma-plot of the vetted data set (omitting formations with more than 10 references contributing to its community matrix before subsampling: lined dots and colored fits). Fits represent loess fit curves with 95% confidence interval in grey.

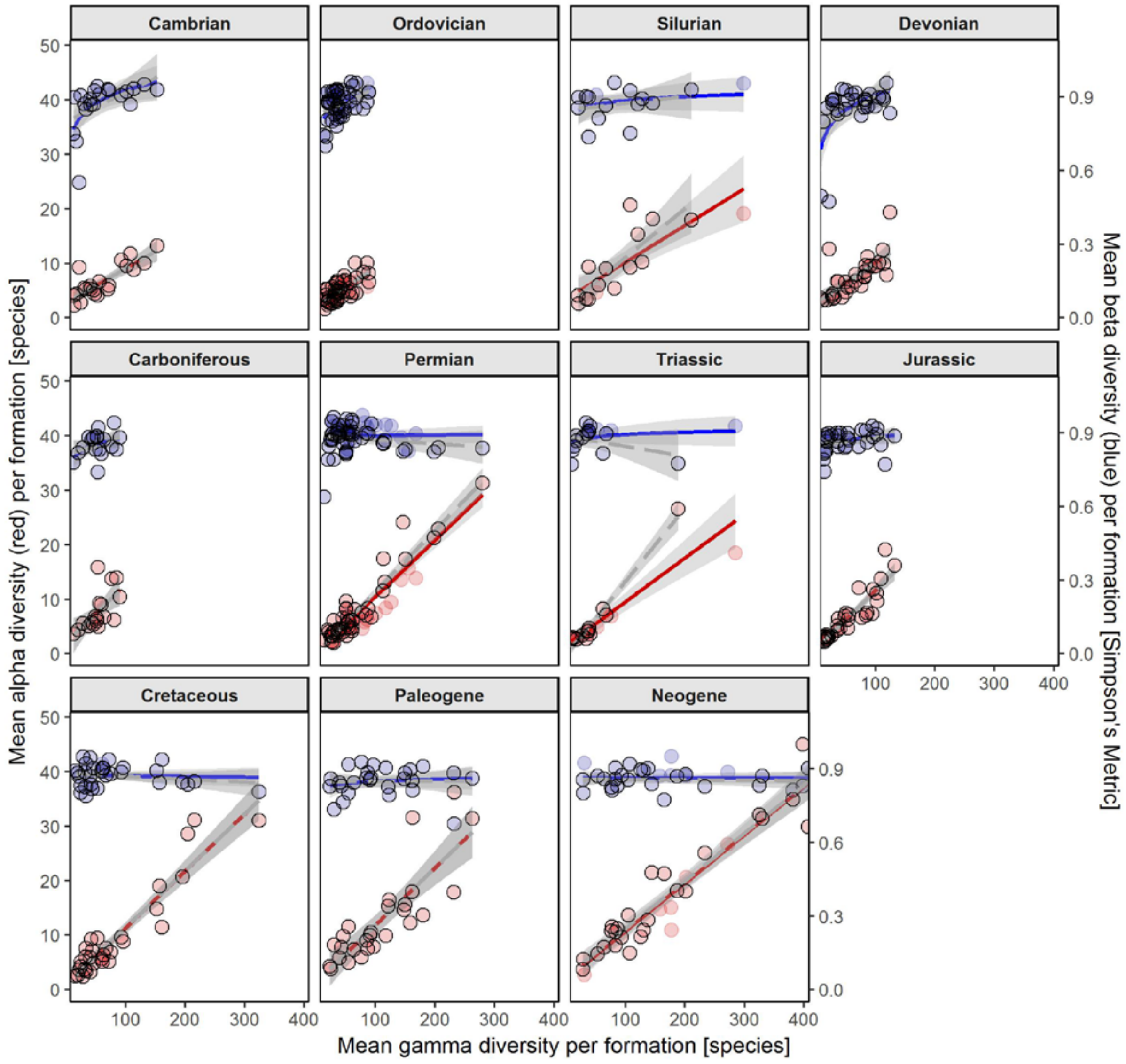

Fig. S10. Comparison of alpha-beta-gamma-plot per period of the full data set (all 340recognized formations: unlined dots and stippled fit) and the vetted data set (omitting formations with more than 10 references contributing to its community matrix before subsampling: lined dots and colored fits) for each formation. Fits with curves with 95% confidence interval in grey.

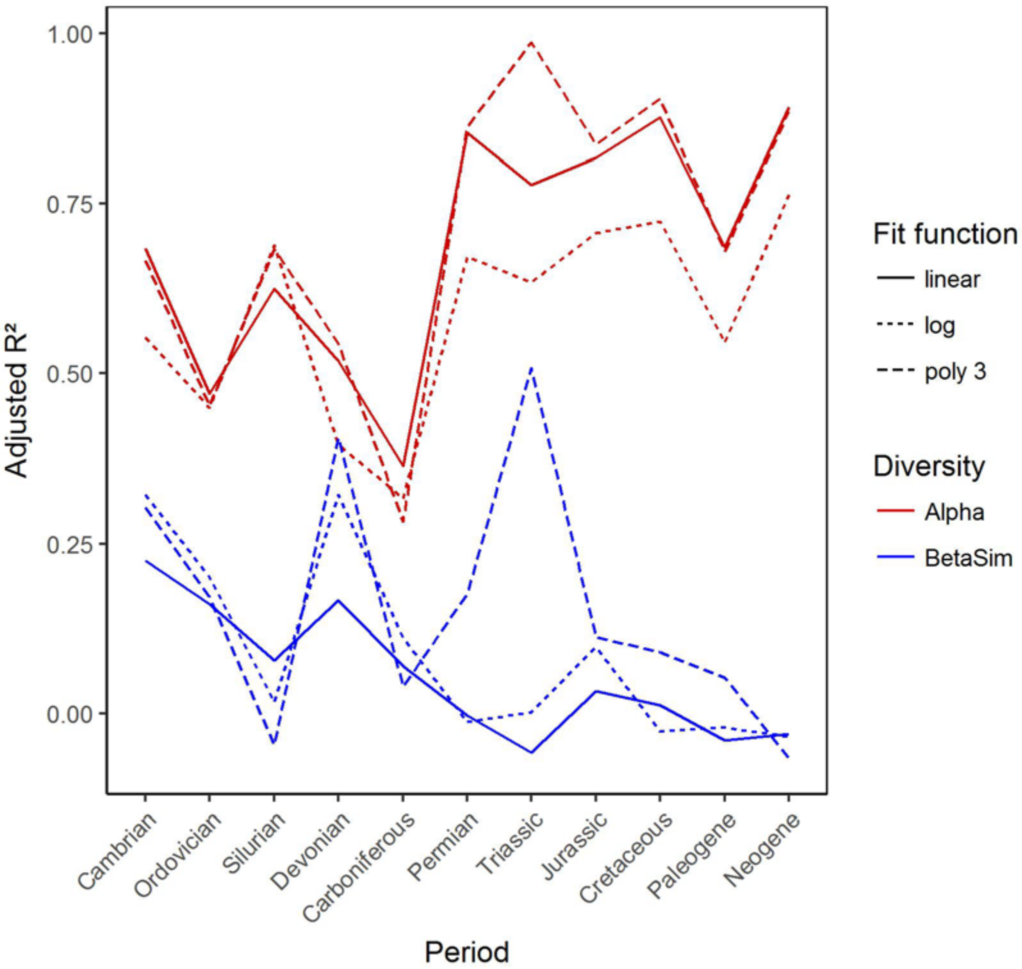

Fig. S11. Summary of R-squared values for various fit functions (blue: beta diversity [BetaSim], red: alpha diversity) presented in Figure 2. Logarithmic fits represent better fits for beta diversity than linear fits and vice versa for alpha diversity. R-squared values of three-term polynomial fits as expected idealized fits are plotted to underscore the adequacy of fits chosen.

Table S1. Results of Spearman rank correlation between variables and diversity. GCD = great circle distance

Variable/Diversities	Gamma		Alpha		BetaW		BetaSim	
	Rho	*p*	Rho	*p*	Rho	*p*	Rho	*p*
collections per formation	0.17	<< 0.01	0.10	0.07	0.15	0.01	0.14	0.01
references per formations	0.41	<< 0.01	0.20	<< 0.01	0.43	<< 0.01	0.33	<< 0.01
duration	0.15	<< 0.01	-0.01	0.85	0.35	<< 0.01	0.36	<< 0.01
number of environments	0.32	<< 0.01	0.20	<< 0.01	0.22	<< 0.01	0.17	<< 0.01
numbers of higher taxa present	0.23	<< 0.01	0.19	<< 0.01	0.08	0.14	0.06	0.29
maximum GCD	0.21	<< 0.01	0.02	0.67	0.38	<< 0.01	0.35	<< 0.01
median GCD	0.21	<< 0.01	0.01	0.83	0.42	<< 0.01	0.37	<< 0.01
median absolute deviation GCD	0.16	<< 0.01	0.01	0.88	0.32	<< 0.01	0.30	<< 0.01
